# Fluorescence-Guided Surgery in Combination with UVC Irradiation Cures Metastatic Human Pancreatic Cancer in Orthotopic Mouse Models

**DOI:** 10.1371/journal.pone.0099977

**Published:** 2014-06-12

**Authors:** Yukihiko Hiroshima, Ali Maawy, Yong Zhang, Sho Sato, Takashi Murakami, Mako Yamamoto, Fuminari Uehara, Shinji Miwa, Shuya Yano, Masashi Momiyama, Takashi Chishima, Kuniya Tanaka, Michael Bouvet, Itaru Endo, Robert M. Hoffman

**Affiliations:** 1 AntiCancer, Inc., San Diego, California, United States of America; 2 Department of Surgery, University of California San Diego, San Diego, California, United States of America; 3 Yokohama City University Graduate School of Medicine, Yokohama, Japan; Wayne State University, United States of America

## Abstract

The aim of this study is to determine if ultraviolet light (UVC) irradiation in combination with fluorescence-guided surgery (FGS) can eradicate metastatic human pancreatic cancer in orthotopic nude–mouse models. Two weeks after orthotopic implantation of human MiaPaCa-2 pancreatic cancer cells, expressing green fluorescent protein (GFP), in nude mice, bright-light surgery (BLS) was performed on all tumor-bearing mice (n = 24). After BLS, mice were randomized into 3 treatment groups; BLS-only (n = 8) or FGS (n = 8) or FGS-UVC (n = 8). The residual tumors were resected using a hand-held portable imaging system under fluorescence navigation in mice treated with FGS and FGS-UVC. The surgical resection bed was irradiated with 2700 J/m^2^ UVC (254 nm) in the mice treated with FGS-UVC. The average residual tumor area after FGS (n = 16) was significantly smaller than after BLS only (n = 24) (0.135±0.137 mm^2^ and 3.338±2.929 mm^2^, respectively; *p* = 0.007). The BLS treated mice had significantly reduced survival compared to FGS- and FGS-UVC-treated mice for both relapse-free survival (RFS) (*p*<0.001 and *p*<0.001, respectively) and overall survival (OS) (*p*<0.001 and *p*<0.001, respectively). FGS-UVC-treated mice had increased RFS and OS compared to FGS-only treated mice (*p* = 0.008 and *p* = 0.025, respectively); with RFS lasting at least 150 days indicating the animals were cured. The results of the present study suggest that UVC irradiation in combination with FGS has clinical potential to increase survival.

## Introduction

Complete tumor resection can improve overall survival of pancreatic cancer patients [1], which is presently 5% at five years. Metastatic relapse often occurs following attempted curative resection of the primary tumor, because all cancer cells are not removed by the surgeon due to the inability to see them. Making tumors fluoresce offers great advantages for tumor detection during surgery to achieve complete resection [2]. We have previously showed that fluorescence-guided surgery (FGS) for pancreatic cancer decreased the residual tumor burden and improved overall and disease-free survival in mouse models [3]–[5]. However, it is difficult to remove all microscopic disease even with FGS [5].

Ultraviolet (UV) light irradiation has shown efficacy in different mouse models in our laboratory in vitro and in vivo on cancer cells expressing fluorescent proteins [6]–[10]. We previously reported UV-induced cancer cell death was found to be wave-length and dose dependent as well as cell-line dependent, with UVC being most effective [9]. In vitro, as little as 25 J/m^2^ UVC irradiation killed approximately 70% of 143B human osteosarcoma cells expressing green fluorescent protein (GFP) and red fluorescent protein (RFP). Cell death began approximately 4 h after irradiation and continued until 10 h after irradiation. UVC exposure also suppressed cancer cell growth in nude mice in a model of minimal residual cancer (MRC) [7], [9]. We also showed that murine Lewis lung carcinoma (LLC) and human U87 glioma cells, expressing GFP in the nucleus and RFP in the cytoplasm, were more sensitive to UVC light than non-colored LLC and U87 cells, suggesting that the expression of fluorescent proteins in cancer cells can enhance the photodynamic effect of UVC on cancer cells.

In the present study, we demonstrated that UVC irradiation used to irradicate MRC after FGS is more effective for human pancreatic cancer in orthotopic mouse models than FGS alone, and results in apparent cures.

## Materials and Methods

### Establishment of Green Fluorescent Protein Labeled Cancer Cell Line

For green fluorescent protein (GFP) gene transduction of cancer cells, 70% confluent MiaPaCa-2 human pancreatic cancer cells [11], [12] were used. In brief, cells were incubated with a 1∶1 precipitated mixture of retroviral supernatants of PT67-GFP cells which express the GFP gene linked to the G418 resistance gene and RPMI 1640 medium (Irvine Scientific, Santa Ana, CA) containing 10% fetal bovine serum (FBS) (Hyclone Laboratories, Logan, UT) for 72 h. Fresh medium was replenished at this time. Cells were harvested with trypsin/EDTA 72 h post-transduction and subcultured at a ratio of 1∶15 into medium, which contained 200 µg/ml of the selective agent G418. The level of G418 was increased stepwise up to 800 µg/ml [11], [13]–[15].

### Cell Culture

MiaPaCa-2-GFP and BxPC-3 [16] human pancreatic cancer cells were maintained in RPMI 1640 medium supplemented with 10% FBS. The cells were incubated at 37°C in a humidified atmosphere of 5% CO_2_ in air. The cells were collected after trypsinization and stained with trypan blue (Sigma-Aldrich, St. Louis, MO). Only viable cells which excluded trypan blue were counted with a hemocytometer (Hausser Scientific, Horsham, PA).

### Animals

Athymic *nu/nu* nude mice (AntiCancer Inc., San Diego, CA), 4–6 weeks old, were used in this study. Mice were kept in a barrier facility under HEPA filtration. Mice were fed with autoclaved laboratory rodent diet. All mouse surgical procedures and imaging were performed with the animals anesthetized by intramuscular injection of a 0.02 ml solution of 50% ketamine, 38% xylazine, and 12% acepromazine maleate. All animal studies were conducted with an AntiCancer Institutional Animal Care and Use Committee (IACUC)-protocol specifically approved for this study and in accordance with the principles and procedures outlined in the National Institute of Health Guide for the Care and Use of Animals under Assurance Number A3873-1.

### Subcutaneous Tumor Cell Implantation

MiaPaCa-2-GFP cells were harvested by trypsinization and washed twice with serum-free medium. Cells (2×10^6^ in 100 µL serum-free medium) were injected subcutaneously within 30 min of harvesting, over the right and left flanks in male nude mice. Subcutaneous tumors were allowed to grow for 2–4 weeks until large enough to supply adequate tumor to harvest for subsequent orthotopic implantation.

### Orthotopic Tumor Implantation

A small 6- to 10-mm transverse incision was made on the left flank of the mouse through the skin and peritoneum. The tail of the pancreas was exposed through this incision, and a single tumor fragment (3-mm^3^) from subcutaneous tumors was sutured to the tail of the pancreas using 8-0 nylon surgical sutures (Ethilon; Ethicon Inc., NJ, USA). On completion, the tail of the pancreas was returned to the abdomen, and the incision was closed in one layer using 6-0 nylon surgical sutures (Ethilon) [17]–[19].

### Fluorescence Imaging

The Olympus OV100 Small Animal Imaging System (Olympus Corp.), containing an MT-20 light source (Olympus Biosystems, Planegg, Germany) and DP70 CCD camera (Olympus Corp., Tokyo, Japan) [20] and the Dino-Lite imaging system (AM4113T-GFBW Dino-Lite Premier; AnMo Electronics Corporation, Taiwan) [21] and the MVX10 long-working-distance microscope (Olympus Corp.) [22], were used for imaging live mice. All images were analyzed with ImageJ v1.440 (National Institutes of Health).

### Tumor Resection and UVC Irradiation

**Figure 1:**
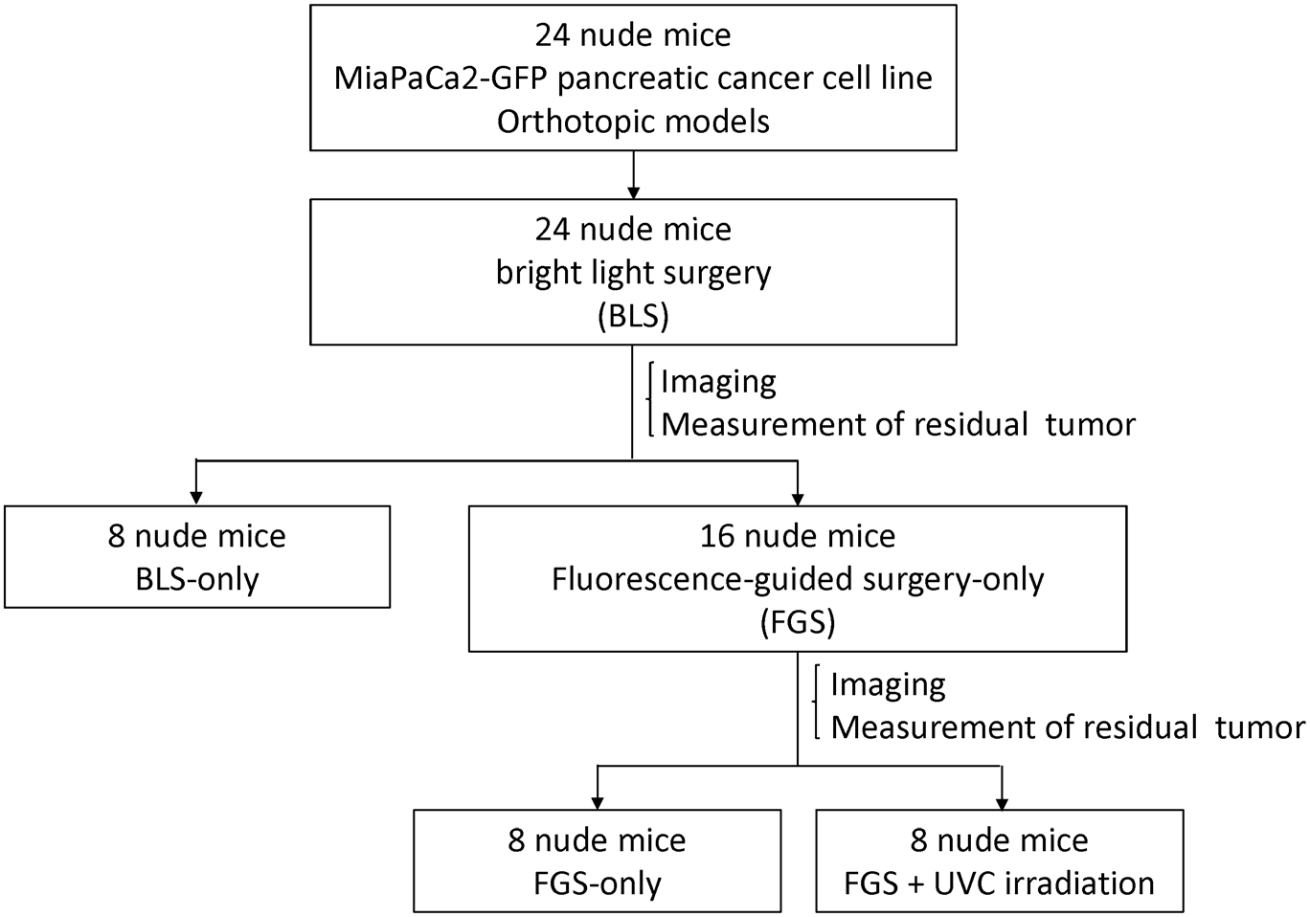
Schematic diagram of the experimental protocol. Two weeks after orthotopic implantation of MiaPaCa-2-GFP pancreatic cancer, bright-light surgery (BLS) was performed on all tumor-bearing mice (n = 24). Postoperatively, the surgical resection bed was imaged with the OV100 at a magnification of 0.56x to detect residual tumor. Mice which underwent BLS were randomized into 3 treatment groups: BLS-only (n = 8), FGS (n = 8), or FGS-UVC (n = 8). Residual tumors in mice in the FGS and FGS-UVC groups were resected using the Dino-Lite imaging system under fluorescence navigation. After completion of FGS, the surgical resection bed was imaged with the OV100 at a magnification of 0.89x to detect micoscopic minimal residual cancer (MRC). The surgical resection bed in the mice in the FGS-UVC group was irradiated with 2700 J/m^2^ UVC (emission peak, 254 nm) from the bottom of the chamber using a Benchtop 3UV transilluminator (UVP, LLC, Upland, CA).

Two weeks after orthotopic implantation of MiaPaCa-2-GFP pancreatic cancer, bright-light surgery (BLS) was performed to all tumor-bearing mice (n = 24). The exposed pancreatic tumor was imaged preoperatively with the OV100 at a magnification of 0.14x. Resection of the primary pancreatic tumor was performed under standard bright-field using the MVX10 microscope. Postoperatively, the surgical resection bed was imaged with the OV100 at a magnification of 0.56x to detect residual tumor. The mice which underwent BLS were randomized into 3 treatment groups: BLS-only (n = 8), FGS (n = 8), or FGS-UVC (n = 8) (Fig. 1). The residual tumors of the FGS or FGS-UVC groups of mice were resected using the Dino-Lite imaging system under fluorescence navigation. After completion of FGS, the surgical resection bed was imaged with the OV100 at a magnification of 0.89x to detect microscopic minimal residual cancer (MRC) [23]. The surgical resection bed of the FGS-UVC group of mice was irradiated with 2700 J/m^2^ UVC (emission peak 254 nm) from the bottom of the chamber using a Benchtop 3UV transilluminator (UVP, LLC, Upland, CA). The incision was closed in one layer using 6-0 nylon surgical sutures. After treatment, the mice were allowed to recover in their cages.

### Noninvasive Imaging of Tumor Recurrence and Progression

To assess for recurrence and to follow tumor progression postoperatively, weekly non-invasive whole-body imaging of the mice was performed with the OV100 at a magnification of 0.14x, until the end of the experiment.

### Monoclonal Antibody

Monoclonal antibodies specific for carcinoembryonic antigen (CEA) were purchased from RayBiotech, Inc. (Norcross, GA). The antibodies were conjugated with the Dylite 488 Protein Labeling Kit (Molecular Probes Inc., Eugene, OR) according to the manufacturers.

### Sensitivity of Non-colored or Fluorescent Pancreatic Cancer Cells to UVC Irradiation in vitro

To compare the efficacy of UVC irradiation on non-colored BxPC-3 or fluorescent pancreatic cancer cells, BxPC-3 cells were labeled with anti CEA antibody conjugated with Dylite 488 (BxPC-3-Ab488) or GFP (BxPC-3-GFP). BxPC-3-GFP or BxPC-3-Ab488 or non-colored BxPC-3 cells (10^3^) were plated in 100 µl cell culture medium per well in 96-well plates. The cells were irradiated with UVC at various doses (0, 25, 50 and 100 J/m^2^) from a Benchtop 3UV transilluminator (UVP, LLC, Upland, CA). Twenty hours after UVC irradiation, the cells were washed with phosphate buffer saline (PBS) three times. The cell number was determined with an IX71 fluorescence microscope (Olympus, Tokyo, Japan).

### Statistical Analysis

PASWStatistics 18.0 (SPSS, Inc) was used for statistical analyses. Residual tumor area is expressed as mean ± SD. The two-tailed Student’s *t*-test was used to compare continuous variables between 2 groups. Kaplan–Meier survival curves were used for estimating survival. Survival outcomes were compared using log rank tests. A *p* value<0.05 was considered statistically significant for all comparisons.

## Results

### FGS Significantly Reduces Tumor Volume but does not Eradicate All Residual Cancer Cells

**Figure 2:**
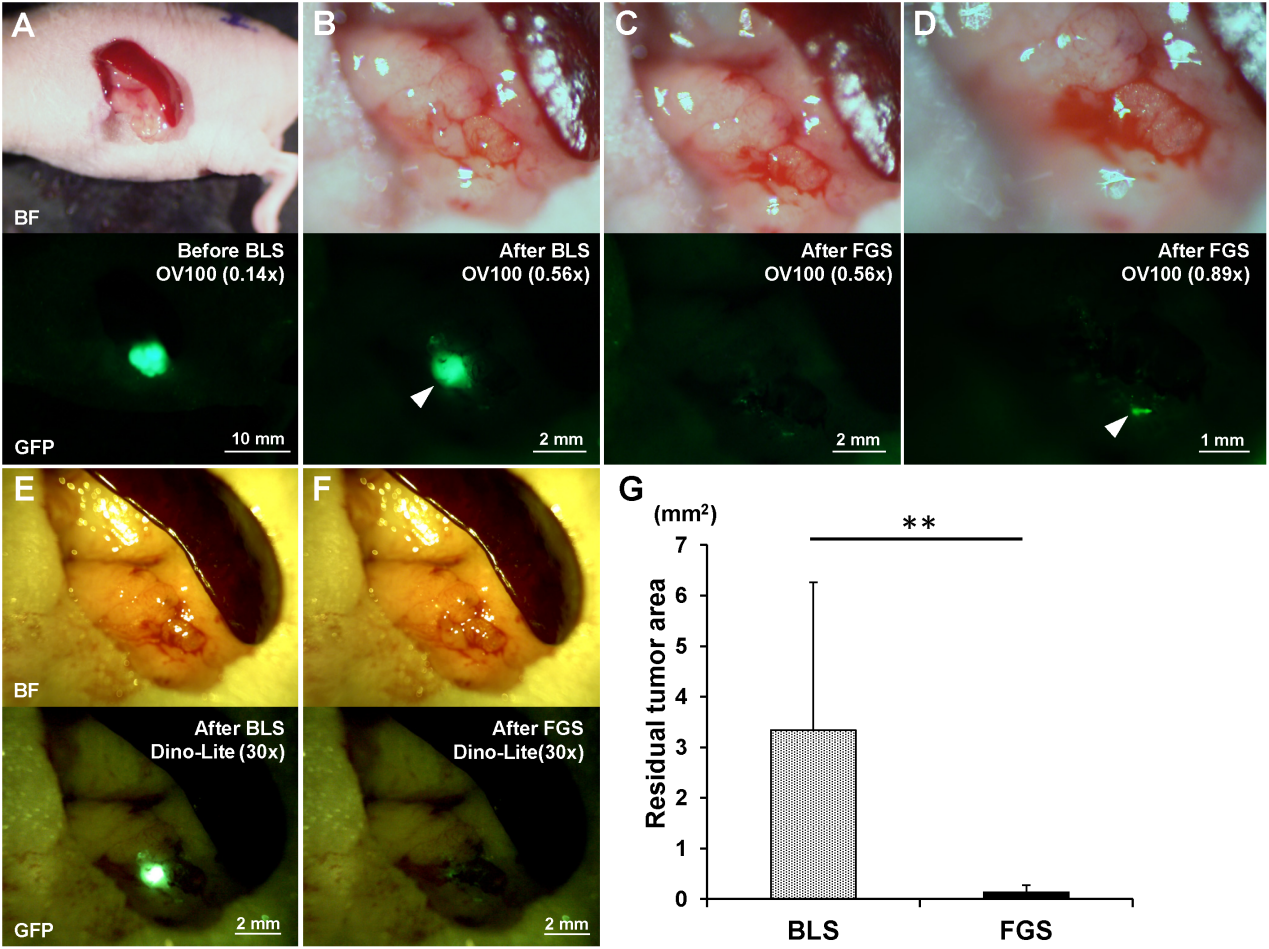
Preoperative and postoperative images of the orthotopic pancreatic cancer model (A–F). Upper panels are bright-field (BF), and lower panels show tumor fluorescence. The residual tumor after BLS was clearly detected with both the OV100 at a magnification of 0.56x (B) and the Dino-Lite at a magnification of 30x (E). The residual tumor after FGS was marginally detected with either the OV100 at a magnification of 0.56x (C) or the Dino-Lite at a magnification of 30x (F). The OV100 at a magnification of 0.89x clearly detected the minimal residual tumor after FGS (D). (G) The residual tumor area after FGS was significantly smaller than after BLS. All images were measured for residual tumor areas using ImageJ. **p<0.01.

BLS was performed on all tumor-bearing mice (n = 24). The exposed pancreatic tumor was imaged preoperatively with the OV100 at a magnification of 0.14x (Fig. 2A). Postoperatively, the surgical resection bed was imaged with the OV100 at a magnification of 0.56x (Fig. 2B) to detect unresected tumor. The mice which underwent BLS were randomized into 3 treatment groups; BLS-only (n = 8), FGS (n = 8), or FGS-UVC (n = 8) (Fig. 1).

Imaging was performed on all 24 animals after BLS and before any other treatment. The average residual tumor area of each group was 3.46±3.45 mm^2^ (BLS), 3.26±2.69 mm^2^ (FGS) or 3.30±2.99 mm^2^ (FGS-UVC). The extent of residual disease was statistically equivalent.

For FGS, the Dino-Lite hand-held portable imaging system was used (Video S1). After completion of FGS, the surgical resection bed was imaged with the OV100 at a magnification of 0.89x (Fig. 2D) to detect microscopic MRC. MRC after BLS-only was clearly detected with both the OV100 at a magnification of 0.56x (Fig. 2B) and the Dino-Lite at a magnification of 30x (Fig. 2E). MRC after FGS was marginally detected with either the OV100 at a magnification of 0.56x (Fig. 2C) or the Dino-Lite at a magnification of 30x (Fig. 2F). The OV100 at high magnification (0.89x) could readily detect microscopic MRC after FGS (Fig. 2D). The average residual tumor area after FGS (n = 16) was significantly smaller than BLS-only (n = 24) (0.135±0.137 mm^2^ and 3.338±2.929 mm^2^, respectively; *p* = 0.007). These results suggest that FGS significantly reduced the residual tumor volume after BLS-only, but microscopic MRC remained on the surgical bed even after FGS.

### UVC Irradiation in Combination with FGS Cures Metastatic Human Pancreatic Cancer

**Figure 3:**
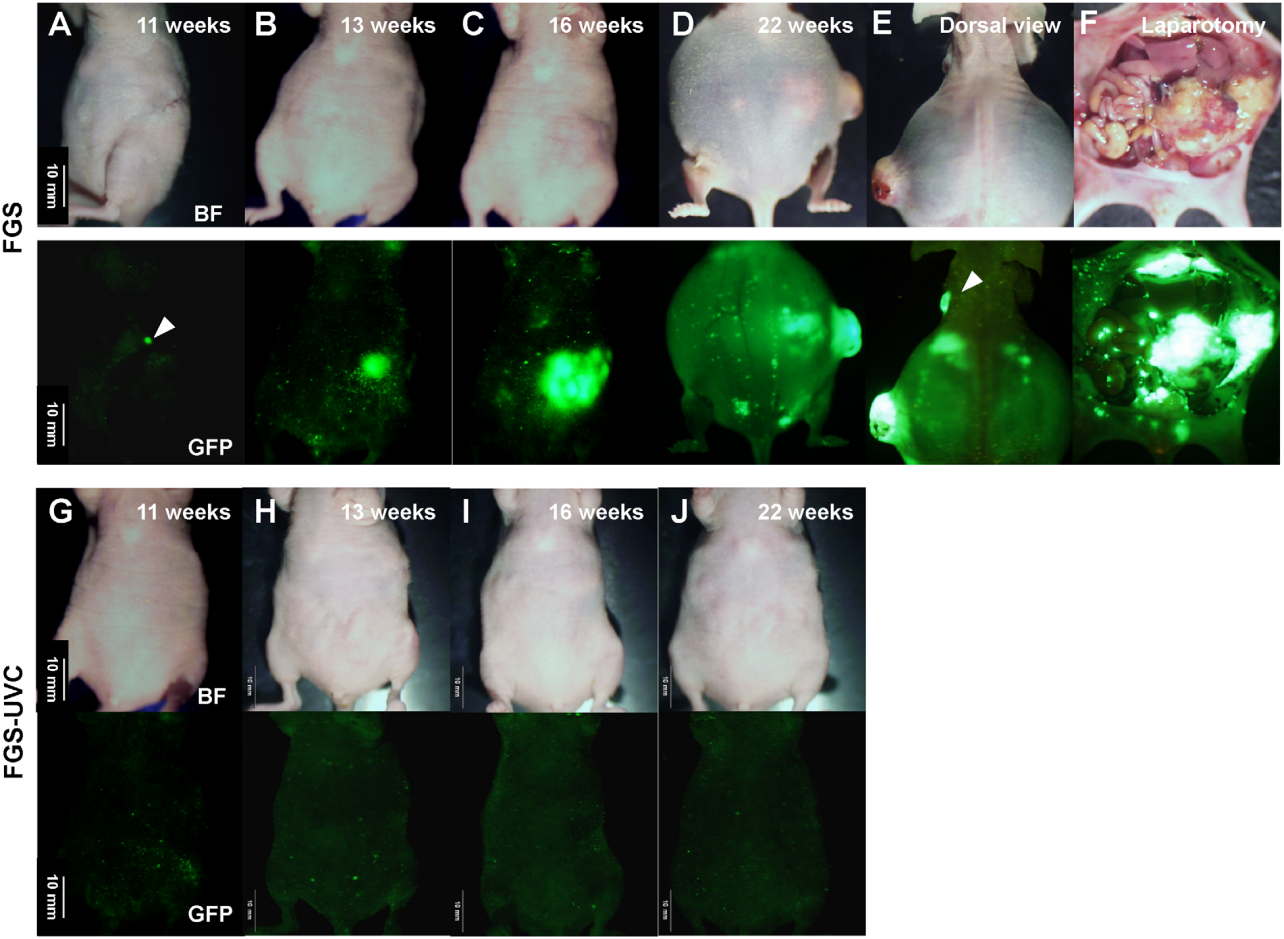
Representative time course of tumor recurrence after FGS. The recurrence was initially detected by non-invasive whole-body imaging using the OV100 at a magnification of 0.14x at week 11 after FGS (A; white arrowhead). The recurrent tumor progressed rapidly (B–D) and killed the mice by week 22 after FGS (D). Left axillary lymph-node metastasis (E; white arrowhead), large local recurrent tumor and many disseminating tumor nodules (F) were detected in the mice at time of death. In contrast, no recurrence was detected in the FGS-UVC group (G–J). Scale bars: 10 mm.

**Figure 4:**
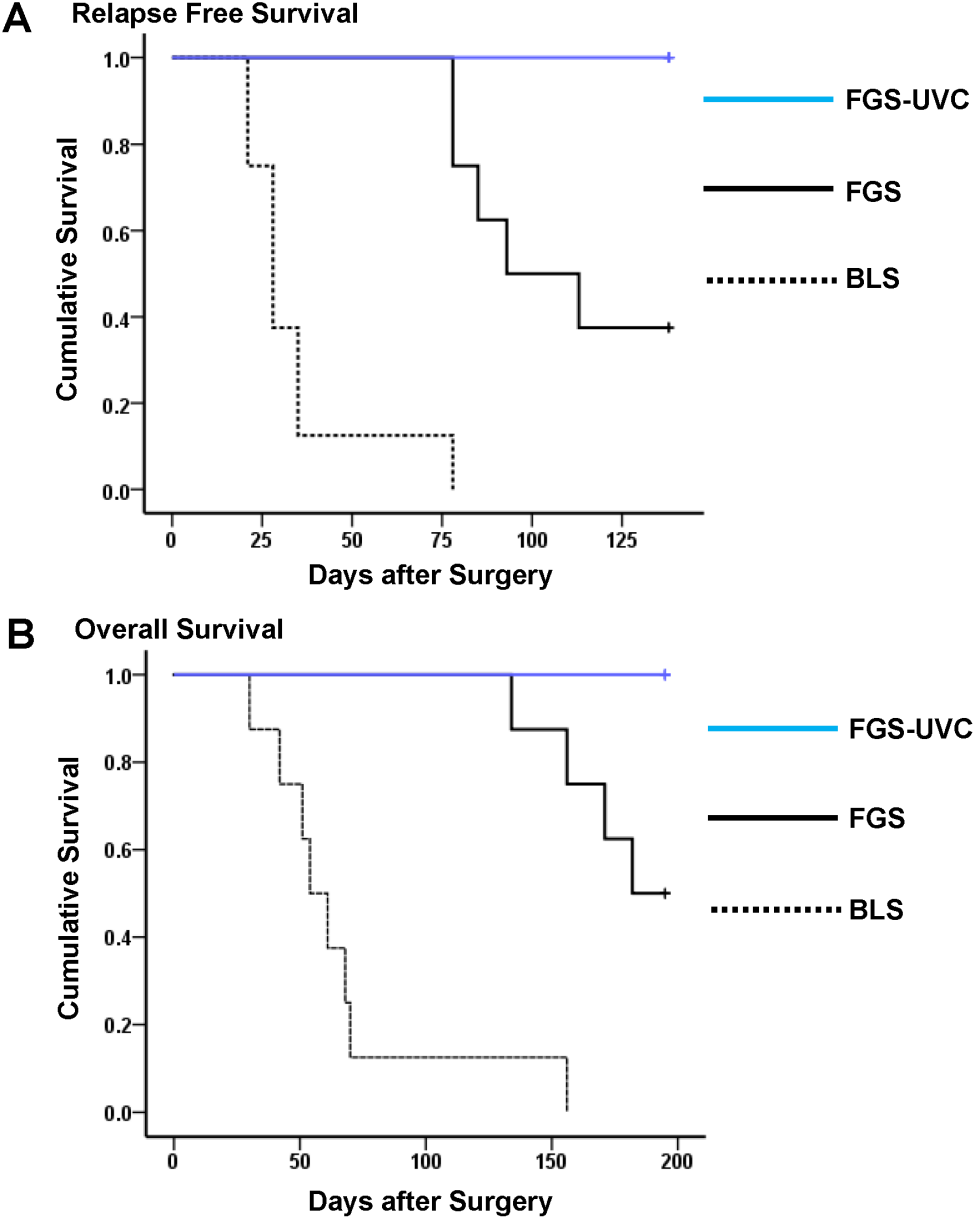
Relapse-free survival (RFS) (A) and overall survival (OS) (B) for tumor-bearing mice treated with BLS-only, FGS, or FGS-UVC.

Recurrent tumors were detected with noninvasive whole-body imaging at weeks 3 to 11 in the BLS-only treated mice and at weeks 11 to 20 in the FGS-only treated mice (Fig. 3A and 4). Recurrence was detected in the 8 mice (100%) in the BLS-only group and 5 mice (62.5%) in the FGS group (Fig. 4). All recurrent tumors progressed rapidly and metastasized regionally and distantly and killed the mice between days 30 to 156 in the mice treated with BLS-only and between days 134 to 195 in the mice treated with FGS (Fig. 3B–3F and 4B). In contrast, no recurrence or death were detected in any mice treated with FGS-UVC (Fig. 3G–3J and 4B), suggesting that UVC irradiation eradicated microscopic MRC after FGS.

### Survival Impact of UVC Irradiation in Combination with FGS

Relapse-free survival (RFS) and overall survival (OS) were estimated in the 3 experimental groups of mice. As shown in Figures 4A and 4B, 3-month RFS in mice after BLS-only, FGS and FGS-UVC was 0%, 50% and 100%, respectively; and 5-month OS was 10%, 90%, and 100%, respectively. Median RFS in mice treated with BLS-only, FGS and FGS-UVC were 28 days, 103 days, and 138 days, respectively, and median OS was 57.5 days, 188.5 days, and 195 days, respectively (Table 1). Mice treated with BLS-only showed significantly reduced survival compared to mice treated with FGS and FGS-UVC for both RFS (*p*<0.001 and *p*<0.001, respectively) and OS (*p*<0.001 and *p*<0.001, respectively). FGS-UVC treated mice showed significantly longer survival compared to mice treated with FGS for both RFS and OS (*p* = 0.008 and *p* = 0.025, respectively) (Fig. 4 and Table 1), suggesting that UVC irradiation in combination with FGS eradicated microscopic MRC.

**Table 1:** Relapse-free survival (RFS) and overall survival (OS) for tumor-bearing mice treated with BLS-only or FGS or FGS-UVC.

Treatment	Median	*p*-value	Median	*p*-value
	RFS (days)	(Log-Rank)	OS (days)	(Log-Rank)
BLS-only	28	–	57.5	–
FGS	103	*p*<0.001*	188.5	*p*<0.001*
FGS-UVC	138	*p*<0.001*, *p* = 0.008**	195	*p*<0.001*, *p* = 0.025**

*compared to BLS.

**compared to FGS.

### Efficacy of UVC Irradiation on Pancreatic Cancer Cells Labeled with Anti-CEA Antibody Conjugated with Dylite 488 *in vitro* or GFP Compared to Unlabeled Cells

**Figure 5:**
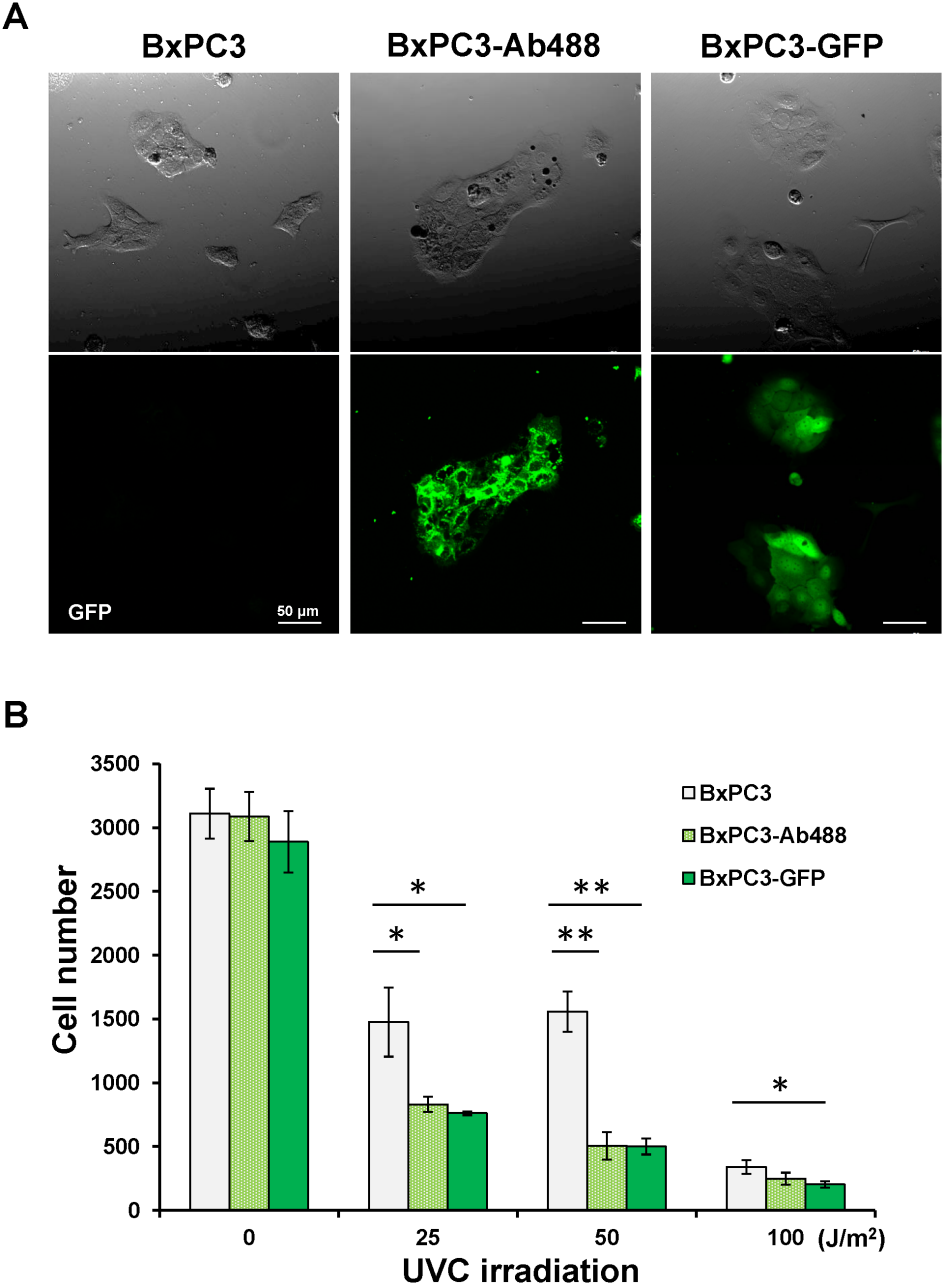
Efficacy of UVC irradiation on non-colored BxPC-3, BxPC-3-Ab488 and BxPC-3-GFP in vitro. (A) Representative images of non-colored BxPC-3, BxPC-3-Ab488 and BxPC-3-GFP in vitro. Cells were observed with the FV1000 confocal microscope (Olympus, Tokyo, Japan). Scale bars: 50 µm. (B) UVC was irradiated at various doses (0, 25, 50 and 100 J/m^2^). Compared to non-colored BxPC-3 cells, the number of BxPC-3-Ab488 and BxPC-3-GFP cells decreased significantly due to UVC irradiation with 25 and 50 J/m^2^. The experimental data are expressed as the mean ± SD. *p<0.05, **p<0.01.

The efficacy of UVC irradiation on BxPC-3 pancreatic cancer cells labeled with anti CEA antibody conjugated with Dylite 488(BxPC-3-Ab488), BxPC-3-GFP and unlabeled BxPC-3 was compared (Fig. 5A). UVC was irradiated at various doses (0, 25, 50 and 100 J/m^2^). Compared to non-colored BxPC-3 cells, the number of BxPC-3-Ab488 and BxPC-3-GFP cells decreased significantly due to UVC irradiation with 25 J/m^2^ (p = 0.016 and p = 0.01, respectively) and 50 J/m^2^ (p = 0.001 and p>0.001, respectively). BxPC-3-Ab488 and BxPC-3-GFP cells were similarly more sensitive to UVC light than non-colored BxPC-3 cells.

## Discussion

The surgical orthotopic implantation (SOI) mouse model used in the present study has been directly compared to clinical outcome of the patient donors in a previous study of ours. Fresh surgical specimens derived from patients with advanced stomach cancer were orthotopically transplanted in nude mice using SOI. There were statistically-significant correlations (p<0.01) for both liver metastases and peritoneal involvement between patients and mice [24].

In another previous study of ours, pancreatic-cancer specimens were transplanted to the nude-mouse pancreas using SOI [25]. The resulting models represented clinical pancreatic cancer including: extension of the locally growing human pancreatic cancer to the nude-mouse stomach and duodenum; metastases to the liver and regional lymph nodes; and distant metastases to the adrenal gland, diaphragm, and mediastinal lymph nodes. The transplanted human pancreatic tumors showed a similar pattern of expression of human tumor-associated glycoprotein 72 and carcinoembryonic antigen.

We have previously shown that SOI of intact human stomach cancer tissue resulted in the formation of metastases in 100% of the mice with extensive primary growth to the regional lymph nodes, liver, and lung. In contrast, orthotopic implantation of cell suspensions of the same human stomach cancer at the same site, metastases occurred in only 6.7% of the mice with local tumor formation. These results emphasize the importance of using SOI to allow full expression of metastatic potential [26].

We also previously compared the metastatic rate of human renal cell carcinoma SN12C when transplanted by SOI or orthotopic implantation of cell suspensions in the kidney. Metastatic rates in the involved organs (lung, liver, and mediastinal lymph nodes) were 2–3 fold higher with SOI compared to cellular orthotopic implantation. Median survival time in the SOI model was 40 days, which was significantly shorter than that of cellular orthotopic implantation (68 days) [27].

In another of our previous studies, we compared SOI to cellular orthotopic transplantation of bladder cancer. After SOI of the RT-10 bladder tumor, metastases occurred in the regional and distant lymph nodes, liver, pancreas, spleen, and tissue adjacent to the adrenal gland and ureter, as well as the lungs. When disaggregated RT-10 cells were injected transurethrally, no metastases formed [28], [29].

With regard to fidelity of drug response, in another previous study of ours, cisplatin (CDDP) had significant efficacy on small-cell lung cancer (SCLC) growing orthotopically in the lung and mitomycin C (MMC) did not, which reflected the clinical situation. In contrast, when the SCLC was growing subcutaneously, the tumors responded to MMC and not to CDDP. These results indicated that the tumors growing orthotopically reflect the clinical effects of drugs on human SCLC more closely than the tumors growing subcutaneously [30]. Therefore, the SOI model should not affect the UV sensitivity of the pancreatic cells as they are growing on their natural orthotopic organ.

A major problem in surgical oncology is MRC after apparent curative tumor resection [23]. We have previously demonstrated the enhanced visualization and resection of primary and metastatic cancer by FGS with the use of telomerase-dependent adenovirus (OBP-401) that expresses the *gfp* gene only in cancer cells which express the telomerase enzyme [31]–[33].

FGS studies have also been previously carried out in our laboratory by labeling the tumors with tumor-specific fluorescent antibodies which have direct clinical applicability [3]–[5], [34]–[36].

A fluorophore-conjugated antibody to CEA was used to evaluate FGS of pancreatic tumors in SOI mouse models of human pancreatic cancer BxPC-3. After intravenous injection of anti-CEA-Alexa Fluor 488, complete resection was achieved in 92% of mice in the FGS group compared to 45.5% in the BLS group. Cure rates with FGS compared to BLS improved from 4.5% to 40%, respectively, and 1-year postoperative survival rates increased from 0% with BLS to 28% with FGS. Median DFS increased from 5 weeks with BLS to 11 weeks with FGS. Median OS increased from 13.5 weeks with BLS to 22 weeks with FGS [37].

The results of these previous studies show the great potential of FGS. However, technical problems remain to remove MRC after FGS. In the present study, we demonstrated that the MRC remained in the surgical bed even after FGS (Fig. 2D), which progressed rapidly and could kill the animals (Fig. 3B–3F). However, UVC irradiation in combination with FGS completely prevented recurrence (Fig. 3G–3J and 4B) and showed significantly increased survival compared to FGS alone for both RFS and OS (*p* = 0.008 and *p* = 0.025, respectively) (Fig. 4 and Table 1). These results suggest that UVC irradiation could eradicate microscopic MRC remaining after FGS.

We have previously shown that UVC irradiation is able to penetrate up to 40 µm in three-dimensional histoculture using Gelfoam and in an *ex vivo* tumor model, as well as kill superficial cancer cells up to a depth of 40 µm *in vivo* without damage of deep tissues [7]. In the present study, MRC was visualized, but only under high magnification, after FGS (Fig. 2D). UVC was able to eradicate the MRC without apparent side effects and eliminated recurrence (Fig. 3G–3J and 4B). These results suggest that a depth of 40 µm is of sufficient depth to eradicate microscopic MRC after FGS. Thus UVC irradiation can sterilize the surgical resection bed of cancer cells after FGS.

Furthermore, we have demonstrated in this study that BxPC-3-Ab488 cells were more sensitive to UVC light than non-colored BxPC-3 cells and equivalent in sensitivity to BxPC-3-GFP cells (Fig. 5B). These results suggest that UVC irradiation could eradicate microscopic MRC, labeled with an exogenous fluorophore, after FGS and that the technology described in the present report is applicable in clinical practice.

## Supporting Information

Video S1 **Resection of the residual tumor after BLS under fluorescence navigation.**
(MP4)
